# Delayed Inflammatory Reaction to Periorbital Hyaluronic Acid Filler: Relapse After Lisinopril Withdrawal and Response to Retreatment

**DOI:** 10.1111/jocd.71281

**Published:** 2026-11-05

**Authors:** Özge Sevil Karstarlı Bakay, Burak Gül, Nida Gelincik Kaçar

**Affiliations:** ^1^ Department of Dermatology Pamukkale University Faculty of Medicine Denizli Türkiye

Dear Editor,

Hyaluronic acid (HA) fillers are widely used for facial and extrafacial rejuvenation and generally well tolerated [1]. Delayed inflammatory reactions (DIRs) may present with edema, erythema, induration, or nodules after an uneventful interval [1]. Their pathogenesis remains uncertain and may involve immune responses, filler degradation, and infectious or biofilm‐related factors; reported triggers include viral infections, sinusitis, dental procedures, different or low‐quality fillers, and injection‐related factors [1]. Individual susceptibility may also contribute, with certain human leukocyte antigen subtypes associated with an increased risk of immune‐mediated filler reactions [2]. DIRs have been reported after severe acute respiratory syndrome coronavirus 2 (SARS‐CoV‐2) infection and coronavirus disease 2019 (COVID‐19) vaccination [3, 4, 5].

A 46‐year‐old woman was referred with suspected angioedema after 2 months of recurrent periorbital swelling, with intermittent improvement and relapse. Her medical history included asthma and hypothyroidism; she was taking levothyroxine 50 μg/day for hypothyroidism. She had received periorbital HA injections 2 years earlier, developed COVID‐19 approximately 18 months after injection, and experienced swelling 6 months after recovery.

Complement C4 and C1 esterase inhibitor levels and routine hematologic and biochemical investigations were normal. Ultrasonography showed hypoechoic deposits compatible with residual HA and surrounding edema, without drainable collection, abscess, or vascular complication. The distribution of swelling and ultrasonographic findings favored filler‐related DIR rather than angioedema. Previous COVID‐19 infection was considered a possible contributing factor.

After discussing treatment options, a short course of systemic corticosteroids was proposed but declined because of concerns about adverse effects. An oral antihistamine was ineffective. Lisinopril, an angiotensin‐converting enzyme inhibitor, was initiated at 10 mg/day, with marked improvement within 1 week. The patient discontinued treatment herself; edema recurred 2 days later. Restarting lisinopril 10 mg/day again improved symptoms. After 1 week, the dose was reduced to 5 mg/day, and treatment was discontinued at the end of the second week (Figures 1 and 2). No recurrence occurred during 6 months of follow‐up.

**FIGURE 1:**
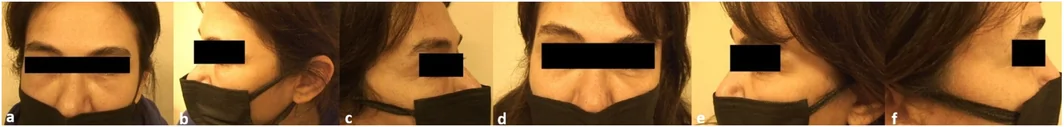
Clinical appearance of the periorbital hyaluronic acid filler reaction before treatment (a–c) and after 1 week of lisinopril 10 mg/day (d–f). Frontal (a, d), left lateral (b, e), and right lateral (c, f) views are shown.

**FIGURE 2:**
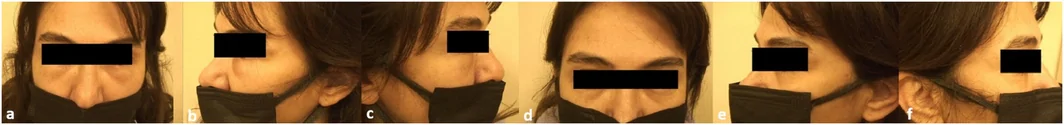
Relapse of the reaction 2 days after lisinopril discontinuation (a–c) and improvement after 1 week of retreatment with lisinopril 10 mg/day (d–f). Frontal (a, d), left lateral (b, e), and right lateral (c, f) views are shown.

Altered cutaneous renin–angiotensin signaling has been proposed in DIR after vaccination and may also be relevant after infection [6]. Spike protein interactions with angiotensin‐converting enzyme 2 may impair local regulation of angiotensin II, promoting cytokine and chemokine release and CD44 expression [6]. CD44 binds extracellular HA, particularly smaller degradation fragments; persistent deposits may therefore recruit inflammatory cells and promote a predominantly CD8^+^/Th1 response [6].

Treatment is not standardized and depends on presentation, severity, and suspected etiology [2]. In their expert‐derived algorithm, Artzi et al. generally favored antibiotics for suspected infectious or biofilm‐related processes, hyaluronidase to reduce residual HA, and systemic corticosteroids mainly for severe, disfiguring, or resistant inflammation [2].

Lisinopril reduces angiotensin II production; reduced aldosterone secretion may promote sodium and water excretion and lessen interstitial edema [6]. These proposed anti‐inflammatory and antiedematous effects could explain our patient's rapid improvement. Based on limited published experience, a starting dose of 5 mg/day, increased to 10 mg/day for an insufficient response, has been proposed [6]. Suggested treatment duration is 3–5 days or at least 1–2 days after swelling subsides [6]. Captopril 6.25 mg three times daily has been proposed as a shorter‐acting alternative [6].

DIR may fluctuate or resolve spontaneously, so a single case cannot establish treatment efficacy. Nevertheless, recurrence after withdrawal and improvement after reintroduction suggest that lisinopril contributed to the clinical response in our patient. The six‐month interval between COVID‐19 recovery and swelling prevents a confident causal attribution, although prior infection may have influenced local immune responses around persistent HA. This case highlights the potential role of ACE inhibition in delayed inflammatory reactions to hyaluronic acid fillers and suggests that recurrence may occur after premature treatment withdrawal. Further studies are needed to clarify the optimal duration of therapy and the mechanisms underlying this response.

## Author Contributions

Özge Sevil Karstarlı Bakay: study conception and design; patient management; critical revision of the manuscript; final approval. Burak Gül: conceptualization; literature review; data collection; data analysis and interpretation; manuscript drafting; manuscript writing; final approval. Nida Gelincik Kaçar: critical revision of the manuscript; final approval.

## Funding

The authors have nothing to report.

## Ethics Statement

Written informed consent was obtained from the patient. Institutional ethical approval was waived due to the nature of a single case report.

## Consent

Written informed consent for publication of clinical details and images was obtained from the patient.

## Conflicts of Interest

The authors declare no conflicts of interest.

## Data Availability

Data sharing is not applicable to this article as no datasets were generated or analysed during the current study.
